# The Types and Consistency of Participant‐Specified Proxies During SPRINT

**DOI:** 10.1002/alz.095415

**Published:** 2025-01-09

**Authors:** Sarah A. Gaussoin, Byron C Jaeger, Alan J. Lerner, Letitia H. Perdue, Stephen R. Rapp, David M. Reboussin, Jeff D. Williamson

**Affiliations:** ^1^ Wake Forest University School of Medicine, Winston‐Salem, NC USA; ^2^ Case Western Reserve University School of Medicine, Cleveland, OH USA

## Abstract

**Background:**

For clinical trials or patient care, reports from a person familiar with the trajectory of a participant’s cognitive and functional performance (i.e., proxies) may improve adjudication of events, such as mild cognitive impairment and dementia. Proxies offer a unique perspective on cognitive status that is different from what is captured through standard cognitive examinations. Clinical trials in which cognitive status is adjudicated, such as the Systolic Blood Pressure Intervention (SPRINT) trial, provide information about the contributions and consistency of proxies throughout extended follow‐up.

**Method:**

SPRINT participants named proxies and relationship to them at study onset. Proxies provided information for the Functional Activities Questionnaire and Dementia Questionnaire. We collapsed proxy relationships into seven categories and evaluated the proportion of proxies in each category at baseline and how frequently the same proxy was retained to the last visit. We explored associations between proxy relationship and various baseline demographics and examined the association between not having a proxy, as well as proxy relationship, and adjudicated outcome.

**Result:**

Of the 9,361 randomized SPRINT participants (35.6% female, mean age 67.9 years), 7,586 (81%) participants listed a proxy relationship from which we identified 7 categories: spouse/partner (51.7%), child (23.6%), sibling (8.7%), other family member (6.2%), and unrelated (8.8%). Male participants were more likely to name a spouse as their proxy (64%) while female participants more likely to name a child (42%). The overall consistency of proxy continuation between the baseline and final SPRINT visit (mean 6.9 years) was 90.7%. Proxy consistency from first visit to last visit for a child was 94.8%, spouse/partner (91.9%) brother/sister (87.5%), other family member (82.4%), non‐related (81.2%), and parent (78.2%). Adjudications that resulted in ‘Cannot Classify’ were greatest for proxies who were parents (15%) followed by none (12%), sibling and grandchild (9%), child and unrelated (7%), spouse and other family (6%).

**Conclusion:**

The majority of proxies in SPRINT were immediate family members (spouse, child, or sibling). Over 90% of identified proxies at baseline continued to be available over a mean of almost 7 years of follow‐up. Not having a proxy available led to a higher rate of ‘cannot classify’ adjudications.

